# Supplementary material for: Complete map of SARS-CoV-2 RBD mutations that escape the monoclonal antibody LY-CoV555 and its cocktail with LY-CoV016
Source: bioRxiv. 2021 Feb 22:2021.02.17.431683. Preprint. [Version 1] doi: 10.1101/2021.02.17.431683 (PMC7924270; doi:10.1101/2021.02.17.431683)
Supplement: 1 [file NIHPP2021.02.17.431683-supplement-1.pdf]

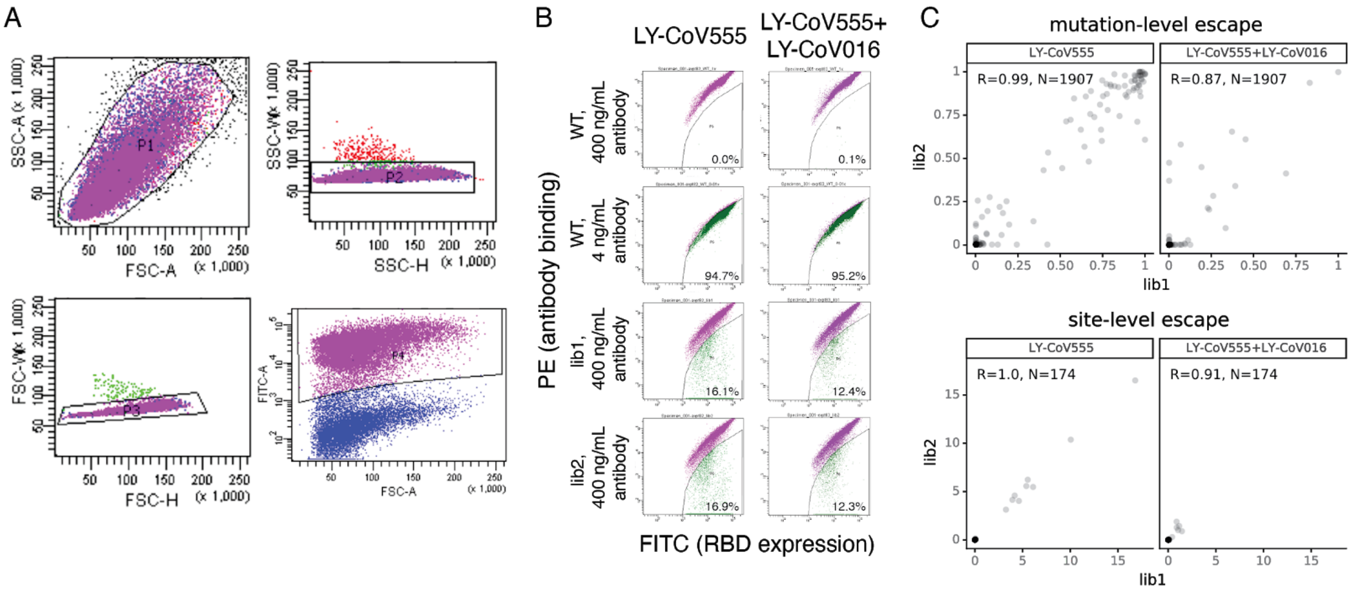

Supplemental Figure 1. Experimental approach for antibody escape mapping. We use fluorescence-activated cell sorting to select yeast mutants that escape antibody binding. We use libraries described by Starr et al. [20] which contain virtually all RBD amino acid mutations, which were previously sorted to select for those compatible with human ACE2 binding as described by Greaney et al. [18]. (A) Initial FACS gates are drawn to select single yeast cells (SSC/FSC, SSC-W/SSC-H, and FSC-H/FSC-W) that properly express RBD (FITC/FSC). (B) Antibody-escape bins are drawn to capture 95% of RBD^{+} cells expressing the unmutated SARS-CoV-2 RBD labeled at 4 ng/mL, indicating 100x reduced binding compared to the 400 ng/mL library selections. The fraction of cells in each population that fall into this “antibody-escape” bin is labeled in each FACS plot. (C) Correlation between independent library duplicates at the level of per-mutation escape fractions (top) or site-level total escape (sum of all mutations at site, bottom).

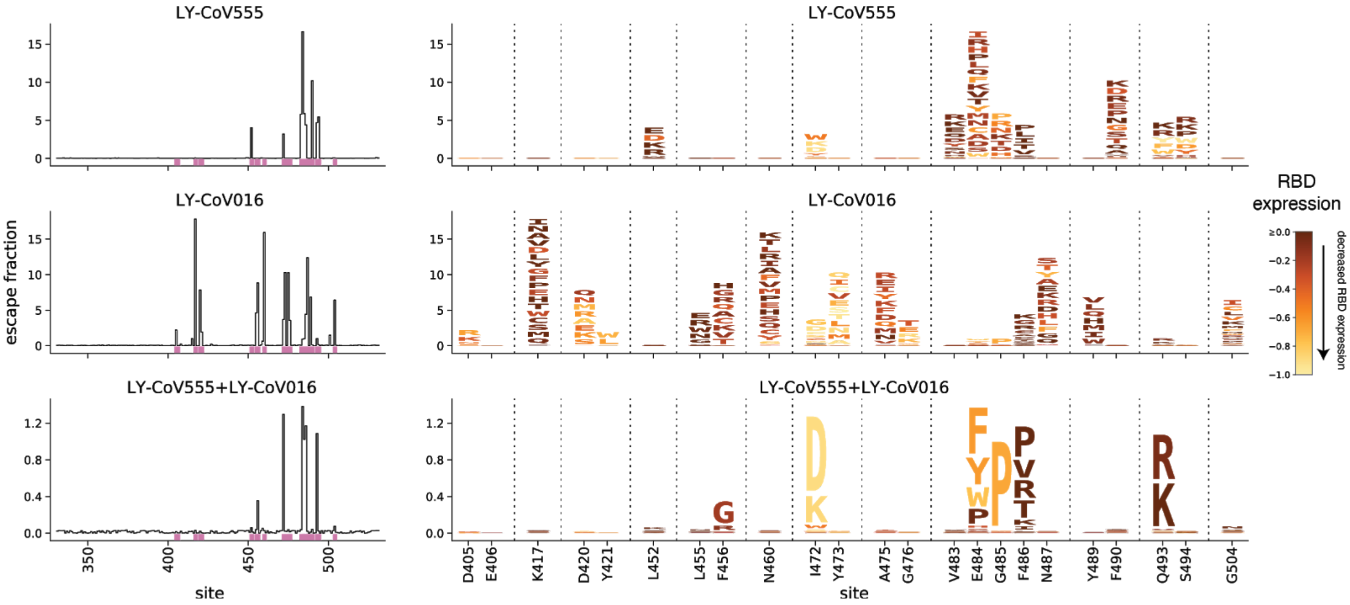

Supplemental Figure 2. Logoplots colored by mutation effects on expression. Line and logoplots are identical to Fig. 1A, except mutations are colored by their effects on the yeast surface expression level of folded RBD, as measured in our previous deep mutational scanning experiment [20].
